# Brain Metastases from Endometrial Carcinoma

**DOI:** 10.5402/2012/581749

**Published:** 2012-03-18

**Authors:** Ettie Piura, Benjamin Piura

**Affiliations:** ^1^Department of Obstetrics and Gynecology, Sapir Medical Center, Sackler School of Medicine, University of Tel-Aviv, Kfar Saba 44281, Israel; ^2^Unit of Gynecologic Oncology, Department of Obstetrics and Gynecology, Soroka Medical Center and Faculty of Health Sciences, Ben-Gurion University of the Negev, Beer Sheva 84101, Israel

## Abstract

This paper will focus on knowledge related to brain metastases from endometrial carcinoma. To date, 115 cases were documented in the literature with an incidence of 0.6% among endometrial carcinoma patients. The endometrial carcinoma was usually an advanced-stage and high-grade tumor. In most patients (*~*90%), brain metastasis was detected after diagnosis of endometrial carcinoma with a median interval from diagnosis of endometrial carcinoma to diagnosis of brain metastases of 17 months. Brain metastasis from endometrial carcinoma was either an isolated disease limited to the brain only (*~*50%) or part of a disseminated disease involving also other parts of the body (*~*50%). Most often, brain metastasis from endometrial carcinoma affected the cerebrum (*~*75%) and was solitary (*~*60%). The median survival after diagnosis of brain metastases from endometrial carcinoma was 5 months; however, a significantly better survival was achieved with multimodal therapy including surgical resection or stereotactic radiosurgery followed by whole brain radiotherapy (WBRT) and/or chemotherapy compared to WBRT alone. It is suggested that brain imaging studies should be considered in the routine follow up of patients with endometrial carcinoma and that the search for a primary source in females with brain metastases of unknown primary should include endometrial biopsy.

## 1. Introduction

The brain, along with the bone, liver, and lung, is one of the most common sites of metastases with about 170,000 patients newly diagnosed with brain metastases each year in USA, a figure which is 10-fold higher than that of patients newly diagnosed with primary brain malignancy [1–3]. Common sources of brain metastases are lung, breast, renal, and colorectal carcinoma and malignant melanoma; it has been estimated that about 15% of patients with these cancers develop brain metastases in the course of their disease [1, 3–5]. Female genital tract cancers, however, are considered “neurophobic” since brain metastases from female genital tract cancers, apart from choriocarcinoma, are rare and usually develop as part of a widespread disseminated disease. The primary mechanism of metastatic spread from genital tract cancers to the brain is by the hematogenous rout. Detached tumor cells are carried from the genital tract by the blood stream through the inferior vena cava, right atrium, right ventricle, pulmonary artery, lungs, pulmonary veins, left atrium, left ventricle, and aorta to the brain [6]. It has been speculated that tumor cells from the genital tract may also travel to the brain via the Batson plexus (paravertebral venous plexus) [3]. Ogawa et al. [6] surveyed 2,729 women with primary genital tract cancers treated during 1985–2006 and identified 18 women (0.7%) with brain metastases. The incidence of brain metastases from ovarian carcinoma (7/335, 2.1%) was higher than those from endometrial carcinoma (4/556, 0.7%), uterine cervix carcinoma (7/1716, 0.4%), and other female genital tract malignancies combined (vagina, vulva, and fallopian tube carcinoma) (0/122, 0%) [6].

 In USA, the number of women newly diagnosed with endometrial carcinoma and the number of women dead of endometrial carcinoma in 2010 has been estimated to be 43,470 and 7,950, respectively [7]. The age-adjusted incidence (number of new cases per 100,000 women per year) and age-adjusted death rate (number of deaths per 100,000 women per year) in 2007 was 24.74 and 4.18, respectively [8]. Endometrial carcinoma in USA is the first most common cancer of the female genital tract, the fourth most common malignancy (after breast, lung, and colorectal cancer) in females accounting for 6% of all cancers in females [7, 8]. It is the eighth most common cause of death from cancer (after lung, breast, colorectal, pancreatic, and ovarian cancer, non-Hodgkin's lymphoma, and leukemia) in females accounting for 3% of all deaths from cancer in females [7]. The National Cancer Institute (NCI) has estimated that the lifetime risk of being diagnosed with endometrial carcinoma and of dying from endometrial carcinoma in females born today in USA is 2.6% (1/38) and 0.5% (1/200), respectively [8].

 In the European Union countries combined (EU-27), the age-standardized incidence of endometrial carcinoma and age-standardized death rate from endometrial carcinoma in 2007 was 16.2 and 3, respectively [9]. In UK, the number of women newly diagnosed with endometrial carcinoma in 2007 was 7,536, and the number of women dead of endometrial carcinoma in 2008 was 1,741 [9]. The age-standardized incidence and death rate in 2008 was 15.7 and 3.1, respectively [9]. Endometrial carcinoma in UK is the first most common cancer of the female genital tract, the fourth most common cancer in women accounting for 5% of all cancers in females [9, 10].

 Worldwide, endometrial carcinoma is the sixth most common cancer (after breast, colorectal, uterine cervix, lung, and gastric cancer) in women with an estimated 287,100 new cases diagnosed in 2008 (4.8% of all cancers in women) and an incidence of 8.2 [11, 12]. Endometrial carcinoma has been estimated to cause 74,000 deaths in 2008 worldwide (2.2% of all cancer deaths in women) with an estimated death rate of 2 [11]. In 2008, the incidence and death rate from endometrial carcinoma was 12.9 and 2.4, respectively, in more developed countries and 5.9 and 1.7, respectively, in less developed countries [12]. Thus, endometrial carcinoma is primarily a cancer of the developed world with incidence rates double those of the less developed countries. Endometrial carcinoma incidence rates are highest in Northern America, up to eight times higher than in parts of Africa [9, 12].

 The vast majority (>90%) of endometrial carcinomas are diagnosed in women aged over 50 years with very few women diagnosed under the age of 35. Incidence after the menopause rises rapidly to a peak of 82 in women in their early seventies [9]. Most (~75%) patients with endometrial carcinoma are diagnosed with an early-stage disease (F.I.G.O. Stage I and II) and the rest (~25%) with an advanced-stage disease (F.I.G.O. Stage III and IV). After primary therapy including total abdominal hysterectomy and bilateral salpingo-oophorectomy followed, if necessary, by adjuvant pelvic radiotherapy, most patients with an early-stage disease will enjoy an overall good prognosis with a 5-year survival rate of about 90%. Nevertheless, even in patients with early-stage disease, survival is lower, and recurrence is more likely with certain adverse features such as high-grade tumors, deep myometrial invasion, unfavorable histologic types, and extension to the uterine cervix [13]. Patients with advanced-stage endometrial carcinoma have a poor prognosis with a median survival often less than one year [13]. The NCI has estimated that endometrial carcinoma at initial diagnosis is found to be localized in the uterus in 70% of the patients (5-year survival rate, 95.5%), with loco-regional extension in 20% of the patients (5-year survival rate, 67.5%) and with distant metastases in 10% of the patients (5-year survival rate, 17.1%) [8]. Although the majority of endometrial carcinoma patients are diagnosed at an early stage with an excellent long-term prognosis, 10–30% of patients develop a recurrent disease [14–16]. Noteworthy, although mortality from endometrial carcinoma declined during the last decades of the twentieth century, there have been reports that endometrial carcinoma incidence and mortality rates are rising in UK and USA [10, 17, 18]. This increase may be attributable to the increase in body mass index in women, but other causes are possible [10].

 Endometrial carcinoma usually spreads by lymphatic vessels to pelvic and para-aortic lymph nodes and by local invasion to the ovaries and surrounding tissues. Less frequently, endometrial carcinoma spreads through the hematogenous pathway. The most frequent sites of hematogenous metastases of endometrial carcinoma are the lungs, including the pleura and mediastinum, liver, and bone [19–21]. There have been several hypotheses explaining the site of preference of distant metastasis from various cancers. Paget [22] proposed in 1889 his “seed and soil” hypothesis, which suggested that specific tumor cell properties and differences in the microenvironment in various organs are responsible for the nonrandom distribution of metastases. Nicolson and Winkelhake [23] suggested in 1975 the possibility that specific cell surface properties of metastasizing tumor cells, and particular properties of the vascular endothelium of the target organs are responsible for the location of distant metastases. This may explain clinical and experimental evidence indicating that circulating tumor cells are present in a wide variety of organs, but implantation of tumor cells occurs only in certain tissues.

 Aalders et al. [24] observed that 379 (11.2%) of 3,393 endometrial carcinoma patients referred to the Norwegian Radium Hospital during 1960–1976 developed recurrent disease. Of the 379 patients with recurrent endometrial carcinoma, 190 (50.1%) had local recurrence, 108 (28.5%) had distant metastases, and 81 (21.4%) had both local recurrence and distant metastases. Sites of distant metastases were the lung: 63 patients, upper abdomen: 48, multiple sites: 26, bone: 22, brain: 11, liver: 10, and lymph nodes: 9. Within one year after completion of primary therapy for endometrial carcinoma, 35% of the local recurrences and 25% of the distant metastases were apparent. Within 3 years, 75% of the local recurrences and 72% of the distant metastases were evident. In about 10% of the patients, however, the recurrence appeared more than 5 years after primary therapy [24]. Sohaib et al. [16] reviewed 86 patients with recurrent endometrial carcinoma following primary surgery and found that disease relapse was detected at an overall median time of 13 months (range, 1–108 months) after primary surgery. Sixty-four percent of relapses were detected within 2 years of surgery, 87% had occurred within 3 years, and 97% within 5 years. The disease recurred locally in 30 (35%) patients (median time to relapse, 11.5 months), distally in 32 (37%) patients (median time to relapse, 20.5 months) and locally and distally in 24 (28%) patients (median time to relapse, 8.5 months). Sites of recurrence were lymph nodes in 41 (48%) patients, vagina: 36 (42%), peritoneum: 23 (27%), lung: 21 (24%), hydronephrosis: 20 (23%), bladder: 7 (8%), liver: 6 (7%), bone: 6 (7%), abdominal wall: 6 (7%), spleen: 4 (5%), rectum: 3 (3%), pancreas: 1 (1%), muscle: 1 (1%), and brain: 1 (1%) (the number of sites of recurrence sums up to more than 86 because a quantity of patients had more than one site of recurrence) [16].

 Brain metastasis from endometrial carcinoma is rare with only 115 patients documented in the literature [16, 19, 21, 24–55] (Table 1). Of 35 papers on brain metastases from endometrial carcinoma published in the literature, 19 documented one patient each [16, 19, 21, 25–29, 32, 38, 40, 43–45, 48, 49, 52, 54, 55], seven: 2 patients each [33–35, 37, 39, 42, 47], two: 3 patients each [30, 50], one: 6 patients [53], one: 8 patients [46], two: 10 patients each [36, 41], two: 11 patients [24, 31], and one: 20 patients [51]. Age at diagnosis of brain metastases from endometrial carcinoma ranged from 40 to 83 years with a median of 60 years (Table 1). Because of the rarity of brain metastases from endometrial carcinoma, the accrual of patients occurred over prolonged periods of time during which treatment approaches and modalities changed. This paper will focus on the following topics related to brain metastases from endometrial carcinoma: characteristics of the primary endometrial carcinoma, brain metastases from endometrial carcinoma in autopsy series, incidence of brain metastases from endometrial carcinoma, interval between diagnosis of endometrial carcinoma and brain metastases, type and site of brain metastases, symptoms and signs of brain metastases, treatment of brain metastases, and survival after diagnosis of brain metastases.

## 2. Characteristics of the Primary Endometrial Carcinoma

The distribution of the F.I.G.O. Stage at diagnosis of endometrial carcinoma in the patients with brain metastases from endometrial carcinoma collated from 35 papers published in the literature [16, 19, 21, 24–55] was as follows: Stage 0 (endometrial intraepithelial neoplasm, EIN): 1 patient, I (not substaged): 4, Ia: 4, Ib: 7, Ic: 4 (Stage I overall: 19), IIa: 2, IIb: 7 (Stage II overall: 9), IIIa: 11, IIIb: 3, IIIc: 18 (Stage III overall: 32), IV (not substaged): 6, IVa: 1, IVb: 11 (Stage IV overall: 18), and not available: 26 (Table 1). Of the 79 patients in whom stage of endometrial carcinoma at initial diagnosis was known, 29 (36.7%) had Stage I + II, and 50 (63.3%) had Stage III + IV. Grade of endometrial carcinoma was G1 in 4 patients, G2: 12, G3: 27, and unknown: 32 (Table 1). Of the 73 patients in whom grade of endometrial carcinoma was known, 4 (5.5%) had G1, 12 (16.4%): G2, and 57 (78.1%): G3. Histological type of endometrial carcinoma was adenocarcinoma in 63 patients, adenosquamous carcinoma: 10, clear cell carcinoma: 4, serous carcinoma: 4, carcinosarcoma: 3, undifferentiated: 3, and unknown: 18 (Table 1). Of the 87 patients in whom histological type of endometrial carcinoma was known, 63 (72.4%) had adenocarcinoma and 24 (27.6%) had other types of endometrial carcinoma (“unfavorable types”). Cormio et al. [36] observed that compared to all patients registered for endometrial carcinoma at their institution (1,069 patients) during 1982–1994, those who developed brain metastases (10 patients) had at the time of diagnosis of the primary tumor a higher percentage of advanced-stage disease (50% versus 16%) and higher percentage of poorly-differentiated tumors (60% versus 23%). Of 10 patients with brain metastases from endometrial carcinoma reported by Mahmoud-Ahmed et al. [41], 9 (90%) had at the time of diagnosis of the primary tumor an advanced-stage disease (Stage III or IV). At the time of diagnosis of the primary endometrial carcinoma in 8 patients with brain metastases reported by Gien at al. [46], 6 (75%) presented with an advanced-stage (Stage III or IV) disease and 5 (62.5%) with a poorly-differentiated (G3) tumor. All 3 patients with brain metastases from endometrial carcinoma documented by Orrù et al. [50] had at the time of diagnosis of the primary disease a poorly-differentiated (G3) advanced-stage (Stage III) endometrial carcinoma. At the time of diagnosis of the primary endometrial carcinoma in 20 patients with brain metastases reported by Chura et al. [51], 17 (85%) presented with an advanced-stage (Stage III or IV) disease, 11 (55%) with a poorly-differentiated (G3) tumor, and 8 (40%) with an unfavorable histologic type (carcinosarcoma, adenosquamous carcinoma, and serous carcinoma).

 Primary therapy for endometrial carcinoma was total abdominal hysterectomy and bilateral salpingo-oophorectomy in 43 patients, total abdominal hysterectomy and bilateral salpingo-oophorectomy and lymphadenectomy: 33, pelvic radiotherapy and chemotherapy: 5, pelvic radiotherapy: 1, no therapy: 5, and unknown: 18 (Table 1). Of the 87 patients in whom primary therapy of the endometrial carcinoma was known, 76 (87.4%) had surgery with at least total abdominal hysterectomy and bilateral salpingo-oophorectomy, 6 (6.9%) had radiotherapy, and 5 (5.7%) had no therapy. In the 76 patients who had primary surgery for the endometrial carcinoma, postoperative adjuvant therapy was pelvic radiotherapy in 42 (55.3%) patients, pelvic radiotherapy, and chemotherapy: 10 (13.2%), chemotherapy: 16 (21%), and no or unknown adjuvant therapy: 8 (10.5%) (Table 1).

 It can be concluded that in most patients who develop brain metastases from endometrial carcinoma, the endometrial carcinoma at the time of diagnosis was an advanced-stage (III/IV) (two-thirds of the patients) and high-grade (G3) (80% of the patients) disease that relapsed despite primary therapy with surgery including total abdominal hysterectomy and bilateral salpingo-oophorectomy followed by adjuvant pelvic radiotherapy.

## 3. Brain Metastases from Endometrial Carcinoma in Autopsy Series

### 3.1. Autopsy of Endometrial Carcinoma Patients

Henriksen [20] surveyed 188 necropsies of endometrial carcinoma patients and revealed 2 (1%) patients with brain metastasis. Berge and Lundberg [56] studied postmortem examinations of 111 patients with endometrial carcinoma and revealed that 40 patients had metastasizing disease; however, no brain metastases were found, but there was one metastases in the meninges.

### 3.2. Autopsy of Patients with Brain Metastases

In a Japanese series of 101 postmortem examinations performed on patients with surgically treated metastatic brain tumors, there was one case of endometrial carcinoma as opposed to 59 cases of lung carcinoma and 4 cases of cervical carcinoma [57].

### 3.3. Autopsy of Patients in General

In 15,000 autopsies reviewed retrospectively at the department of pathology, Innsbruck University Hospital, Innsbruck, Austria, there was one case of endometrial carcinoma with brain metastases [58].

## 4. Incidence of Brain Metastases from Endometrial Carcinoma

### 4.1. Incidence of Brain Metastases among Endometrial Carcinoma Patients (Table 1)

Aalders et al. [24] found 11 patients with brain metastases from endometrial carcinoma managed at the Norwegian Radium Hospital, Oslo, Norway between 1960 and 1976, accounting for 0.3% of 3,393 patients with endometrial carcinoma and 2.9% of 379 patients with recurrent endometrial carcinoma. De Porre and Tjokrowardojo [31] reviewed 2,293 patients with endometrial carcinoma treated at the Dr. Daniel den Hoed Cancer Center, Rotterdam, The Netherlands during 1965–1988 and found 11 (0.48%) patients with brain metastases. Cormio et al. [36] found 10 (0.9%) patients with brain metastases among 1,069 endometrial carcinoma patients treated at the Ospedale S. Gerardo, Monza, Italy during 1982–1994. The risk of developing brain metastases was quite low for both patients with advanced-stage endometrial carcinoma (2/64, 3%) and recurrent endometrial carcinoma (8/143, 6%). However, brain metastasis was seen in 8 (29.6%) of 27 patients with distant recurrent disease [36]. Gien et al. [46] reviewed 1,295 patients with endometrial carcinoma referred to the London Regional Cancer Centre University of Western Ontario, Canada during 1991–2003 and found that 8 (0.6%) of these women developed brain metastases. Sohaib et al. [16] reviewed 86 patients with recurrent endometrial carcinoma following primary surgery managed at the Royal Marsden Hospital, London, UK during 1996–2004 and found one patient (1.16%) with brain metastases. Chura et al. [51] found 20 (0.97%) patients with brain metastases among 2,063 endometrial carcinoma patients treated at the University of Minnesota, Women's Cancer Center, Minneapolis, USA during 1995–2006. In six reports in which the number of endometrial carcinoma patients is available, 10,199 endometrial carcinoma patients were surveyed overall and 61 (0.59%) of them were found to have brain metastases [16, 24, 31, 36, 46, 51].

### 4.2. Incidence of Endometrial Carcinoma as the Source of Brain Metastases among Patients with Brain Metastases (Table 1)

Mahmoud Ahmed et al. [41] revealed that endometrial carcinoma was the source of brain metastases in 10 (0.7%) of 1,391 patients with brain metastases treated in their institution during 1982–1999. Orrù et al. [50] found that endometrial carcinoma was the source of brain metastases in 3 (0.9%) of 348 patients with brain metastases referred to the Businco Regional Oncological Hospital, Cagliari, Italy for palliative radiotherapy during 1999–2005. Le Chevalier et al. [59] reviewed retrospectively 120 consecutive patients presenting to The Gustave-Roussy Institute, Villejuif, France during 1959–1979 with brain metastases as the first sign of their malignancy. The primary malignancy was identified in 62 patients (53 alive and 6 dead of disease); endometrial carcinoma was the source of brain metastases in one (1.6%) patient [59]. Overall, thus, endometrial carcinoma was the source of brain metastases in 14 (0.7%) of 1,801 patients with brain metastases.

## 5. Interval between Diagnosis of Endometrial Carcinoma and Brain Metastasis

The interval between diagnosis of endometrial carcinoma and brain metastasis was available in 95 of 115 patients with brain metastases documented in the literature (Table 1). In 82 (86.3%) of these 95 patients, brain metastasis was detected after diagnosis of endometrial carcinoma with an interval between diagnosis of endometrial carcinoma and brain metastasis ranging from 2 to 108 months (median, 17 months). In 4 (4.2%) of the 95 patients, brain metastasis was diagnosed simultaneously with endometrial carcinoma [28, 30, 44, 49]. In 9 (9.5%) of the 95 patients, brain metastasis was detected before the diagnosis of endometrial carcinoma; in 4 patients brain metastasis was detected 0.25 [34], 0.5 [47], 2.5 [30], and 3 months [30], respectively, before the diagnosis of endometrial carcinoma and in 5 patients [35, 41, 42] brain metastasis was detected at unknown time before the diagnosis of endometrial carcinoma. Thus, although in the vast majority of patients (~90%) brain metastasis from endometrial carcinoma is detected late in the course of the primary disease, in some patients (~10%) brain metastasis is detected simultaneously with the primary disease and sometimes even before the detection of the primary disease. Kottke-Marchant et al. [30] showed that invasive, high-grade endometrial carcinoma with vascular invasion may metastasize very early in the course of disease, even before clinical symptoms related to the primary tumor become apparent. Therefore, it has been recommended that the study of brain metastasis of unknown primary site should include endometrial biopsy [38].

## 6. Type and Site of Brain Metastasis

Type of brain metastasis with respect to whether the metastasis is confined to the brain only (isolated brain metastasis) or is part of a disseminated disease was available for 98 patients (Table 1). Of these 98 patients, 48 (49%) had metastasis confined to the brain only, and 50 (51%) had brain metastasis as part of a disseminated disease that affects also extracranial sites. Among patients with concomitant extracranial metastatic disease at diagnosis of brain metastasis, the most common sites of extracranial recurrence were the pelvis, peritoneum, lung, bone, liver, and lymph nodes [36, 41, 46, 51]. Interestingly, half of the patients with brain metastasis from endometrial carcinoma documented in the literature had metastasis confined to the brain only (isolated brain metastasis). Of 10 patients with brain metastases from endometrial carcinoma reported by Cormio et al. [36], 6 (60%) had isolated brain metastases (no other sites of disease) and survived after diagnosis of brain metastases for a median of 3 months (range, 1–83 months) whereas 4 (40%) had brain metastases as part of a disseminated disease and survived for a median of 1 month (range, 1-2 months). Of 10 patients with brain metastases from endometrial carcinoma reported by Mahmoud-Ahmed [41], 3 (30%) had isolated brain metastases and survived after diagnosis of brain metastases for a median of 6 months (range, 1.5–11.5 months) whereas 7 (70%) had brain metastases as part of a disseminated disease and survived for a median of 2.5 months (range, 0.25–15 months). Of 8 patients with brain metastases from endometrial carcinoma reported by Gien et al. [46], 2 (25%) had isolated brain metastases and survived after diagnosis of brain metastases for a median of 4.5 months (range, 2–7 months) whereas 6 (75%) had brain metastases as part of a disseminated disease and survived for a median of 3.5 months (range, 0.25–7 months). Of 20 patients with brain metastases from endometrial carcinoma reported by Chura et al. [51], 4 (20%) had isolated brain metastases and survived after diagnosis of brain metastases for a median of 31.5 months (range, 9.2–39.2 months) whereas 16 (80%) had brain metastases as part of a disseminated disease and survived for a median of 1.1 months (range, 0.1–10.9 months).

 Amount of brain metastases with respect to whether the metastasis is single (solitary) brain metastases or multiple brain metastases was available for 88 patients (Table 1). Of these 88 patients, 50 (56.8%) patients had single (solitary) brain metastases, and 38 (43.2%) patients had multiple brain metastases. Of 10 patients with brain metastases from endometrial carcinoma reported by Cormio et al. [36], 6 (60%) had single brain metastases and survived after diagnosis of brain metastases for a median of 2.5 months (range, 1–83 months) whereas 4 (40%) had multiple brain metastases and survived for a median of 1 month (range, 1–3 months). Of 10 patients with brain metastases from endometrial carcinoma reported by Mahmoud-Ahmed [41], 3 (30%) had single brain metastases and survived after diagnosis of brain metastases for a median of 15 months (range, 11.5–15.5 months) whereas 7 (70%) had multiple brain metastases and survived for a median of 2.5 months (range, 0.25–6 months). Of 8 patients with brain metastases from endometrial carcinoma reported by Gien et al. [46], 4 (50%) had single brain metastases and survived after diagnosis of brain metastases for a median of 3 months (range, 0.5–7 months) whereas 4 (50%) had multiple brain metastases and survived for a median of 3.5 months (range, 0.25–7 months). Of 20 patients with brain metastases from endometrial carcinoma reported by Chura et al. [51], 8 (40%) had single brain metastases, 4 (20%) had 2 brain metastases, 7 (35%) had ≥3 brain metastases, and 1 (5%) had leptomeningeal disease. There was no significant difference in survival between patients with 1 or 2 brain metastases (median survival, 3.1 months) and patients with ≥3 brain metastases (median survival, 2.1 months) (*P* = 0.58) [51].

 Site of metastasis in the brain with respect to whether the metastasis is supratentorial (cerebrum) or infratentorial (cerebellum) or both was available for 66 patients (Table 1). Of these 66 patients, 48 (72.7%) patients had supratentorial metastasis, 10 (15.2%) patients had infratentorial metastasis, and 8 (12.1%) patients had both supratentorial and infratentorial metastasis. This is in contrast to Delattre et al. [60] who observed in 1988 that substantial fraction (50%) of solitary (single) brain metastases from primary pelvic and abdominal tumors is located infratentorially (posterior fossa, cerebellum). The relatively higher frequency of infratentorial metastasis from primary pelvic and abdominal tumors compared to other primary tumors was explained by the role of Batson's paravertebral venous plexus [60]. Nevertheless, the role of Batson's paravertebral plexus in directing metastatic tumor cells from the pelvis and abdomen to the infratentorial region of the brain is dubious since there is no concomitant higher frequency of spine and skull metastases from primary pelvic and abdominal tumors compared to other primary tumors [38]. Of 10 patients with brain metastases from endometrial carcinoma reported by Cormio et al. [36], 7 (70%) had metastases located in the cerebrum and survived after diagnosis of brain metastases for a median of 3 months (range, 1–83 months), 1 (10%) had metastases located in the cerebellum (survival, 3 months), and 2 (20%) had metastases located in both the cerebrum and cerebellum (survival, 1 month). Of 10 patients with brain metastases from endometrial carcinoma reported by Mahmoud-Ahmed [41], 8 (80%) had metastases located in the cerebrum and survived after diagnosis of brain metastases for a median of 2.4 months (range, 0.25–15.5 months), 1 (10%) had metastases located in the cerebellum (survival, 15 months), and 1 (10%) had metastases located in both the cerebrum and cerebellum (survival, 6 months). Of 8 patients with brain metastases from endometrial carcinoma reported by Gien et al. [46], 4 (50%) had metastases located in the cerebrum and survived after diagnosis of brain metastases for a median of 1.25 month (range, 0.25–7 months), 2 (25%) had metastases located in the cerebellum and survived for a median of 3 months (range, 1–5 months), and 2 (25%) had metastases located in both the cerebrum and cerebellum and survived for a median of 6 months (5–7 months).

 Thus, it can be concluded that brain metastasis from endometrial carcinoma is situated in the cerebral parenchyma in almost 75% of the patients and is solitary (single brain metastases) in almost 60% of the patients. Brain metastasis from endometrial carcinoma is an isolated metastatic disease confined to the brain alone in half of the patients and part of a disseminated disease that affects also extracranial sites in the other half of the patients.

## 7. Symptoms and Signs of Brain Metastases from Endometrial Carcinoma

Symptoms and signs of brain metastases from endometrial carcinoma are not different from symptoms and signs of other brain space occupying lesions. In a series of 10 patients with brain metastases from endometrial carcinoma reported by Cormio et al. [36], headache was present in 8 patients, motor weakness: 5, seizures: 2, confusion, dizziness, balance, and visual disturbances: 1 patient each. In 10 patients with brain metastases from endometrial carcinoma reported by Mahmoud-Ahmed [41], loss of consciousness, seizures, motor weakness, and headache were the most common symptoms. All 10 patients reported by Gien et al. [46] developed neurological symptoms, primarily motor dysfunction, speech difficulty, and mental confusion. In 3 patients reported by Orrù et al. [50], aphasia developed in 2 patients, memory loss: 1, hemiparesis: 1, and headache: 1, and temporary loss of consciousness: 1. In a series of 20 patients with brain metastases from endometrial carcinoma reported by Chura et al. [51], the most common neurologic symptoms were confusion: 9 (45%) patients, gait disturbance: 8 (40%), paralysis: 4 (20%), speech difficulty: 2 (10%), and nausea and vomiting: 2 (10%). In the singular case reports of brain metastases from endometrial carcinoma published in the literature, common presenting symptoms and signs of brain metastases included headache, confusion, dizziness, decreased mental status, consciousness disturbance, general weakness, extremity weakness, gait disturbance, neurological motor deficit, hemiparesis, ataxia, visual disturbance, papilledema, incontinence, nausea, vomiting, speech impairment (aphasis), parasthesias, syncope, and seizure.

 In the vast majority of cases, it was the emergence of one of more neurological symptoms and signs in endometrial carcinoma patients that provoked a search for brain metastases with use of brain-imaging studies, most often brain-computed tomography (CT) and less often magnetic resonance imaging (MRI) or both CT and MRI. In many cases, the lesions demonstrated on CT and MRI were associated with brain edema [36]. The brain edema due to the growth of metastases in the brain parenchyma causes increased intracranial pressure which is the main reason for the headache, nausea, vomiting, and papilledema. In the largest series of patients with brain metastases from endometrial carcinoma published in the literature (20 patients), Chura et al. [51] showed that the diagnosis of brain metastases was established with both CT and MRI in 8 (40%) patients, MRI alone in 8 (40%) patients, and CT alone in 4 (20%) patients.

## 8. Treatment of Brain Metastases from Endometrial Carcinoma

Data with respect to treatment modality of brain metastases was available for 91 patients (Table 1). Of these 91 patients, 10 (11%) patients had surgery (craniotomy, resection of brain metastases) alone, 21 (23.1%): surgery followed by whole-brain radiotherapy (WBRT), 7 (7.7%): surgery followed by WBRT and chemotherapy, 1 (1.1%): surgery followed by stereotactic radiosurgery (SRS) and chemotherapy, 8 (8.8%): SRS alone, 26 (28.5%): WBRT alone, 1 (1.1%): WBRT followed by SRS, 5 (5.5%): chemotherapy and WBRT, 1 (1.1%): oral poly(ADP)-ribose polymerase (PARP) 1 inhibitor (Olaparib) alone, 7 (7.7%): steroids alone, and 4 (4.4%): no treatment. Thus, overall, 60 (65.9%) patients had WBRT, 39 (42.8%): craniotomy, 13 (14.3%): chemotherapy, and 10 (11%): SRS (number of patients adds up to more than 91 since some patients had more than one treatment modality). Craniotomy usually consisted of gross total resection of the brain metastases and sometimes of subtotal resection of the brain metastases. As a rule, WBRT consisted of external megavoltage photonic irradiation employing a 10 MeV linear accelerator delivering a total dose of 30 Gray (3,000 RAD) to the whole brain in daily fractions (3 Gray per fraction for 10 fractions).

 In the series of 10 patients with brain metastases from endometrial carcinoma reported by Cormio et al. [36], brain metastasis was treated by craniotomy alone in 1 patient (developed lung metastases after 2 months for which she received cisplatin-based chemotherapy and still alive at last followup), craniotomy followed by WBRT in 2 patients (died after 28 and 83 months, resp.), WBRT alone in 1 patient (died after 3 months) and steroids alone in 6 patients (all died within 1 month of diagnosis). Of note, in 7 of the 9 patients who died, the cause of death was attributed to progressive brain metastasis [36]. The authors conclude that surgical resection of the brain metastases followed by WBRT is considered the best option in patients with solitary and/or resectable metastases in presence of control of systemic disease.

 In the series of 10 patients with brain metastases from endometrial carcinoma reported by Mahmoud-Ahmed et al. [41], brain metastasis was treated by WBRT in 5 (50%) patients (one of them had SRS + WBRT), craniotomy alone in 2 (20%) patients, and craniotomy followed by WBRT in 3 (30%) patients (in one of them craniotomy was followed by SRS + WBRT). Thus, overall, WBRT was given to 8 (80%) patients, SRS was used in 2 (20%) patients, and craniotomy was performed in 5 (50%) patients (number of patients adds up to more than 10 since some patients had more than one treatment modality). The authors [41] observed that the 3 patients who had multimodal therapy with craniotomy followed by WBRT ± SRS had a longer survival (median, 15 months; range, 11.5–15.5 months) than the 5 patients who had WBRT without craniotomy (median. 2.4 months; range, 0.25–6 months) and the 2 patients who had craniotomy alone (median, 2.75 months; range, 1.5–4 months).

 Chura et al. [51] reviewed 20 patients with brain metastases from endometrial carcinoma treated at the University of Minnesota, Women's Cancer Center, Minneapolis, USA during 1995–2006 and found that 7 (35%) patients had WBRT alone, 4 (20%): WBRT followed by chemotherapy (cisplatin and doxorubicin: 2 patients, carboplatin: 1 patient, thalidomide: 1 patient), 1 (5%): craniotomy followed by WBRT, 1 (5%): WBRT followed by SRS, 3 (15%): WBRT, craniotomy and chemotherapy (cisplatin and doxorubicin: 2 patients), topotecan: 1 patient), and 4 (20%): no treatment. Thus, overall, WBRT was the cornerstone of treatment for all 16 (80%) patients who had therapy for brain metastases, and chemotherapy was given to 7 (35%) patients, craniotomy was performed in 4 (20%) patients, SRS was performed in 1 (5%) patient, and no treatment was given to 4 (20%) patients (the number of patients adds up to more than 20 since some patients had more than one treatment modality). The authors [51] showed that multimodal therapy of brain metastases (WBRT + craniotomy, WBRT + craniotomy + chemotherapy, WBRT + chemotherapy) resulted in a longer survival (median, 9.2 months) compared to WBRT alone (median, 0.9 months) (*P* = 0.0001) or no treatment (median, 0.2 months) (*P* = 0.009).

 In studies of brain metastasis from endometrial carcinoma published in the literature, patient accrual occurred over prolonged periods of time during which treatment approaches and modalities for brain metastases changed. Traditionally, patients with isolated (limited to the brain only) and single (solitary) brain metastases generally would undergo resection of the brain metastases by craniotomy followed by WBRT. Patients with multiple brain metastases (with or without extracranial disease) generally would receive WBRT alone. In series and singular case reports published in literature before 2001, stereotactic radiosurgery (SRS) or gamma-knife radiosurgery (GKRS) was yet not included in the treatment of brain metastases from endometrial carcinoma. Petru et al. [42] described in 2001 two patients in whom brain metastases were detected prior to diagnosis of primary endometrial carcinoma. The brain metastases in both patients were treated by SRS. One of these patients died of disease 15 months after diagnosis of brain metastases. The other patient was still alive at the time of writing the report; with no evidence of disease, 171 months after she had SRS for a solitary (single) brain metastases and aggressive cytoreductive abdominal and pelvic surgery followed by doxorubicin-based chemotherapy for the endometrial carcinoma [42]. Shiohara et al. [44] reported in 2003 a woman who underwent the removal of a single brain metastases and one month later had hysterectomy for poorly-differentiated endometrioid endometrial carcinoma. After hysterectomy, a brain magnetic resonance imaging (MRI) demonstrated multiple brain metastases, and the patient had two rounds of SRS and six courses of chemotherapy. Complete response of the brain lesions was obtained, and the patient was well without recurrence 38 months after the second SRS [44]. Monaco et al. [53] evaluated in 2008 the outcomes after SRS for brain metastases in 6 patients with endometrial carcinoma and 21 patients with ovarian carcinoma. Of the 27 patients (results were not stratified according to type of primary tumor), one patient was still alive at the time of last followup, and 26 had died. The median survival was 7 months after the diagnosis of brain metastases and 5 months after SRS. Interestingly, on final imaging studies, all tumors were controlled without further growth; nevertheless, 2 patients (7.4%) developed new or progressive neurologic deficits after SRS. The authors [53] concluded that SRS provides local tumor control with limited morbidity and is an acceptable choice for the treatment of brain metastases resulting from endometrial and ovarian carcinoma; however, careful patient selection is needed in patients with uncontrolled systemic disease in whom a limited survival benefit is expected.

 Although the experience of using SRS in the treatment of brain metastases from endometrial carcinoma is still limited, it seems that SRS will gain more popularity in the future since patients with a limited number of brain metastases may be treated with SRS without WBRT. Repeat SRS may be performed for new lesions in the brain to avoid or delay use of WBRT for as long as possible. The rationale for this approach is the avoidance of significant neurotoxicity from WBRT. Patients treated with SRS alone may experience fewer cognitive and constitutional side effects. In addition, there is an advantage for the use of SRS in treating patients with single brain metastases who are unable to tolerate surgery and for those with surgically inaccessible lesions. Thus, it seems that the role of SRS in the treatment of brain metastases from endometrial carcinoma is expected to increase.

## 9. Survival after Diagnosis of Brain Metastases from Endometrial Carcinoma

The median survival after diagnosis of brain metastases of 10 patients with brain metastases from endometrial carcinoma reported by Cormio et al. [36] was 1 month (range, 1–83 months). The median survival after diagnosis of brain metastases of 10 patients reported by Mahmoud-Ahmed et al. [41] was 3.25 months (range, 0.25–15.5 months). The 8 patients with brain metastases reported by Gien et al. [46] survived after diagnosis of brain metastases for a median of 3.5 months (range, 0.25–7 months). The median survival after diagnosis of brain metastases of 20 patients reported by Chura et al. [51] was 2 months (range, 0.1–39.2 months). The 27 patients (6 endometrial carcinoma patients and 21 ovarian carcinoma patients) reported by Monaco et al. [53] survived from the time of treatment of brain metastases with SRS for a median of 5 months (range, 0.2–25 months).

 The survival time after diagnosis of brain metastases from endometrial carcinoma was available in 91 of 115 patients documented in the literature and ranged from 0.1 to 171 months with a median of 5 months (Table 1). In 40 patients in whom metastasis was confined to the brain only (isolated brain metastases) and survival time was available, the median survival after diagnosis of brain metastases was 12.75 months (range, 0.25–171 months). In 44 patients in whom brain metastasis was part of a disseminated disease and survival time was available, the median survival after diagnosis of brain metastases was 2.22 months (range, 0.1–37 months). In 40 patients who had single brain metastases and in whom survival time was available, the median survival after diagnosis of brain metastases was 12.75 months (range, 0.5–171 months). In 25 patients who had multiple brain metastases and in whom survival time was available, the median survival after diagnosis of brain metastases was 2.5 months (range, 0.25–64 months). In 45 patients with supratentorial (cerebral) metastases and in whom survival time was available, the median survival after diagnosis of brain metastases was 7 months (range, 0.25–171 months). In 10 patients with infratentorial (cerebellar) metastases and in whom survival time was available, the median survival after diagnosis of brain metastases was 5.75 months (range, 1–17 months). In 8 patients with both supratentorial and infratentorial metastases and in whom survival time was available, the median survival after diagnosis of brain metastases was 2.5 months (range, 1–8 months). In 28 patients who had for their brain metastases multimodal therapy including surgery followed by WBRT and in whom survival time was available, the median survival after diagnosis of brain metastases was 22 months (range, 2.1–84 months). In 8 patients who had surgery alone for their brain metastases and in whom survival time was available, the median survival after diagnosis of brain metastases was 2.25 months (range, 1–18 months). In 26 patients who had WBRT alone for their brain metastases and in whom survival time was available, the median survival after diagnosis of brain metastases was 2 months (range, 0.25–17 months).

 Thus, generally, the survival of patients after diagnosis of brain metastases from endometrial carcinoma is poor. Nevertheless, the survival after diagnosis of brain metastases is affected by the status (controlled versus uncontrolled) and extent (cranial metastases only versus cranial and extracranial metastases) of the primary disease and by the number, volume, and site of metastases in the brain parenchyma. A relatively longer survival is achieved with multimodal therapy including craniotomy (or SRS) followed by WBRT ± chemotherapy than by WBRT alone.

## 10. Conclusion

Brain metastasis from endometrial carcinoma is rare with approximately 100 cases documented in the literature. The incidence of brain metastases among patients with endometrial carcinoma is 0.6%. In 0.7% of patients with brain metastases, the source of brain metastases is endometrial carcinoma. The primary endometrial carcinoma metastasizing to the brain is usually an advanced-stage (III/IV) and high-grade (G3) endometrial carcinoma that was treated primarily by total abdominal hysterectomy and bilateral salpingo-oophorectomy followed by adjuvant pelvic radiotherapy. In the vast majority of patients (~90%), brain metastases are detected after diagnosis of endometrial carcinoma (“metachronous metastases”) with a median interval from diagnosis of endometrial carcinoma to diagnosis of brain metastases of 17 months. In some patients (~10%), however, brain metastases are diagnosed at the time of diagnosis of endometrial carcinoma (“synchronous metastases”) or before the diagnosis of endometrial carcinoma. Thus, although brain metastasis from endometrial carcinoma is usually considered a late event in the course of the primary disease, poorly-differentiated endometrial carcinomas with lymph-vascular and deep myometrial invasion may metastasize early in the course of the disease, even before clinical symptoms of the primary tumor become apparent. Brain metastasis from endometrial carcinoma is either an isolated disease limited to the brain only (~50% of the patients) or part of a disseminated disease with metastases occurring also in other sites of the body (~50% of the patients). Most often, brain metastasis from endometrial carcinoma is located in the cerebrum (75% of the patients) and is solitary (single brain metastases) (60% of the patients). The following variables affect the prognosis and guide treatment decisions in patients with brain metastases from endometrial carcinoma: age and performance status of the patient, state of control of the primary disease, absence or presence of extracranial metastases, the number, location, and volume of brain metastases, and mode of therapy of brain metastases. Treatment of brain metastasis has evolved over the years from WBRT only for most patients to multimodal therapy including surgical resection, if feasible, followed by WBRT and/or chemotherapy. The median survival after diagnosis of brain metastases from endometrial carcinoma is 5 months overall; nevertheless, a better median survival is achieved with multimodal therapy including craniotomy followed by WBRT (22 months) compared to craniotomy alone (2.25 months) or WBRT alone (2 months). The experience of using SRS in the treatment of brain metastases from endometrial carcinoma is still limited. Early detection of brain metastases in endometrial carcinoma patients is of utmost importance since brain metastases at their early stage of development in the brain parenchyma are still of small volume and, thus, much more feasible for surgical resection or SRS with less complications and better survival than metastases at advanced stage of their development. The emergence of one of more neurological symptoms and signs in an endometrial carcinoma patient should incite an immediate search for brain metastases with the use of brain-imaging studies. Moreover, it is suggested that brain-imaging studies should be considered in the routine followup of patients with primary endometrial carcinoma. It is also suggested that the search for a primary source in females with brain metastases of unknown primary should include endometrial biopsy.

## Figures and Tables

**Table 1:** Patients with brain metastases from endometrial carcinoma documented in the literature.

Author (year) study period (Ref.)	No. of patients BM/EC	Age at BM	Stage (FIGO) of EC	Grade of EC	Hist of EC	Primary therapy for EC	Adj. therapy for EC	Interval EC to BM (mon.)	Other MTS	No. of BM	Site of BM	Therapy for BM	Survival after BM (mon.)
Salibi and Beltaos (1972) [25]	1	63	NA	NA	AC	HSO	NR	6	No	Single	Supra	S	AW 18
Nakano and Schoene (1975) [19]	1	77	NA	NA	CC	HSO	RT	26	Yes	Single	Supra	S + RT	D 4
Turner and Graf (1982) [26]	1	83	NA	G3	AC	HSO	NR	NA	NA	Single	Supra	S	D 1
Aalders et al. (1984) 1960–1976 [24]	11/3,393	11 NA	11 NA	11 NA	11 NA	11 NA	11 NA	11 NA	8 No	11 NA	11 NA	11 NA	11 NA
	(0.3%)								3 Yes				
Ritchie et al. (1985) [27]	1	61	IIIc	NA	AC	HSO	RT	NA	Yes	Mul.	Supra	NA	D 4
Savage et al. (1987) [28]	1	70	I	G1	AC	HSO	RT	0	Yes	Single	Supra	RT	D 14
Sawada et al. (1990) [29]	1	43	IIIc	G3	AC	HSOL	RT	2	No	Single	Supra	S + RT	AW 84
Brezinka et al. (1990) [21]	1	59	Ic	G2	AC	HSO	No	2	Yes	Single	Supra	S	D 1
Kottke-Marchant (1991) 1980–1988 [30]	3	59	IIIc	G3	CC	HSO	C	− 3	Yes	Single	Supra	S + RT	D 37
		43	IIIa	G3	AC	HSO	No	0	No	Mult.	Both	S	D 1
		46	IIIa	G3	AC	HSO	C	−2.5	No	Single	Supra	S + RT	AW 9
De Porre and Tjokrowardojo (1992) 1965–1988 [31]	11/2,293	67	11 NA	11G3	1AS	HSOL	RT	8	1 No	Single	Supra	S	D 14
	(0.48%)				10 NA	10 NA	10 NA	10 NA	10 NA	10 NA	10 NA	10 NA	10 NA
Thomas and Lambert (1992) [32]	1	51	I	NA	AC	HSO	RT	24	No	Single	Supra	S + RT	AW84
Wronski et al. (1993) [33]	2	70	NA	NA	AC	HSO	RT + C	22	Yes	Mult.	Infra	S + RT	D 5.5
		60	NA	NA	AC	HSO	RT	88	Yes	Mult.	Both	RT	D 2
Ruelle et al. (1994) [34]	2	64	NA	G3	AC	HSO	RT + C	14	Yes	Single	Infra	S + RT	D 9
		63	I	G3	AC	HSO	RT	−0.25	No	Single	Supra	S + RT	AD 24
De Witte et al. (1996) [35]	2	67	0	NA	AC	HSO	No	24	No	Single	Supra	S + RT	D 60
		40	IIIc	G3	AS	HSOL	C	-UK	No	Single	Supra	S + RT	NA
Cormio et al. (1996) 1982–1994 [36]	10/1,069 (0.9%)	59 (47–71)	3 Ib 2 Ic	6 G3	8 AC	5 HSO	8 RT 2 C	26 (3–81)	4 yes	6 Single	7 Supra	1 S	1 (1–83)
				4 NA	1 AS	4 HSOL			6 no	4 mult.	2 Both	2 S + RT	
			2 IIIa		1 CC	1 NA					1 Infra	1 RT 6 Steroids	
			1 IIIc										
			2 IV										
Salvati et al. (1998) [37]	2	48	Ia	G3	AC	HSO	RT	10	No	Single	Supra	S + RT + C	D 20
		54	Ia	G3	AC	HSO	RT	26	No	Single	Supra	S + RT + C	D 74
Martínez-Mañas et al. (1998) [38]	1	76	IIb	NR	AC	HSO	RT	18	No	Single	NA	S	D 8
Ogawa et al. (1999) [39]	2	43	IIb	G2	AC	HSOL	RT	36	Yes	Mult.	Supra	RT	D 5
		64	IIb	G3	AC	HSOL	RT	18	Yes	Mult.	Both	RT	D 3
Crispino et al. (2000) [40]	1	57	Ic	G3	NA	HSOL	RT	12	No	Single	Infra	S + RT	D 3
Mahmoud-Ahmed et al. (2001) 1985–1999 [41]	10^a^	51 (48–80)	1 IIa	10 NA	7 AC	2 HSOL	2 RT	8 (0.25–70)	7 yes	3 Single	8 Supra	5 RT	3.25
			1 IIIa		3AS	4HSO	4 RT + C		3 no	7 Mult.	1 Both	2 S	(0.25–15.5)
			2 IIIb			4 RT + C	4 No	2-UK			1 Infra	3 S + RT	
			2 IIIc										
			4 IV										
Petru et al. (2001) [42]	2	59	IVa	G3	AC	HSO	C	-UK	No	Single	Supra	SRS	AW 171
		60	IIIc	G3	SC	HSO	C	-UK	No	Single	Infra	SRS	D 15
Sewak et al. (2002) [43]	1	63	Ib	G3	AC	HSOL	RT	48+	Yes	Single	Infra	S + RT	D 6
Shiohara et al. (2003) [44]	1	48	IIIa	G3	AS	HSOL	No	0	No	Single	Supra	S + SRS + C	AW 38
Elliot et al. (2004) [45]	1	51	IIb	G3	AC	HSOL	RT	2	No	Single	Supra	S + RT + C	AW 30
Gien et al. (2004) 1991–2003 [46]	8/1,295 (0.6%)	67.5	2IIb	3 G2	4 AC	3 HSO	5 RT	8.5 (0–40)	6 yes	4 Single	4 Supra	6 RT	3.5
		(48–82)	4IIIc	5 G3	2SC	3HSOL	2 C		2 no	4 Multi.	2 Both	1 C + RT	(0.25−7+)
			2IVb		1CC	1 RT	1 NA				2 Infra	1 Steroids	
					1AS	1 R + C							
Salvati et al. (2004) [47]	2	62	Ia	G2	AC	HSO	RT	48	No	Single	Supra	S + RT	AW 9
		51	IIIc	G3	AC	HSO	RT + C	−0.5	Yes	Single	Supra	S + RT + C	D 34
Lee et al. (2006) [48]	1	54	Ib	G3	AC	HSOL	RT	108	No	Mult.	Supra	RT	D 0.25
Llaneza-Coto et al. (2006) [49]	1	43	IIa	G3	AC	No	No	0	No	Single	Supra	S	D 1
Orrù et al. (2007) 1999–2005 [50]	3^b^	61	IIIc	G3	AC	HSO	C + RT	17	No	Mult.	Supra	S + RT	AW 64
		60	IIIa	G3	AC	HSO	No	6	No	Single	Supra	RT	D 4
		49	IIIb	G3	AC	HSO	RT	10	No	Single	Supra	S + RT	AW 16
Sohaib et al. (2007) [16]	1/86^c^ (1.16)	NA	NA	NA	AC	HSO	NA	NA	No	Single	NA	NA	NA
													
Chura et al. (2007) 1995–2006 [51]	20/2,063 (0.97%)	64 (49–78)	1 Ia	3 G1	11 AC	14 HSOL	5 RT	11.5 (0.6–73.6)	16	8 Single	NA	7 RT	2 (0.1–39.2)
			2 Ib	6 G2	3 CS	2 HSO	7C		Yes	12 Mult.		4 RT + C	
			4 IIIa	11 G3	2 AS	4 No	2 RT + C		4 No			1 S + RT	
			4 IIIc		1 SC		6 No					1 RT + SRS	
			9 IVb		3 UD							3S + RT + C	
												4 No	
Ramirez et al. (2008) [52]	1	61	IIb	G3	AC	HSO	RT	12	No	Mult.	Infra	RT	D 17
Monaco et al. (2008) [53]	6	60.4	6 NA	6 NA	6 NA	6 NA	6 NA	6 NA	6 NA	6 NA	6 NA	6 SRS	5 (0.2–25)
Al-Mujaini et al. (2008) [54]	1	69	NA	NA	AC	HSO	RT	84	Yes	Mult.	Supra	NA	NA
Forster et al. (2011) [55]	1	58	I	NA	AC	HSO	RT	108	Yes	Mult.	Supra	Olaparib^d^	D18

Total	115												5 (0.1–171)

AC: adenocarcinoma; Adj: adjuvant; AD: alive with disease; AS: adenosquamous carcinoma; AW: alive and well; BM: brain metastases; C: chemotherapy; CC: clear-cell carcinoma; CS: carcinosarcoma; D: dead of disease; EC: endometrial carcinoma; Hist: histology; Infra: infratentorial (cerebellum); HSO: total abdominal hysterectomy and bilateral salpingo-oophorectomy; HSOL: HSO and lymph node dissection; mon: months; MTS: metastases; Mult: multiple; NA: not available; S: surgery (craniotomy); SC: serous carcinoma; SRS: stereotactic radiosurgery; Supra: supratentorial (cerebrum); UD: undifferentiated, -UK: brain metastases detected at unknown time before the diagnosis of endometrial carcinoma.

^a^ Endometrial carcinoma was the source of brain metastases in 10 (0.7%) of 1,391 patients with brain metastases.

^b^Endometrial carcinoma was the source of brain metastases in 3 (0.9%) of 348 patients with brain metastases.

^c^ Patients with recurrent endometrial carcinoma following primary surgery.

^d^ Oral poly(ADP)-ribose polymerase (PARP) 1 inhibitor.
